# General practitioners’ experiences in consultations with foreign language patients after the introduction of a user’s fee for professional interpretation: a qualitative interview study

**DOI:** 10.1186/s12875-022-01718-7

**Published:** 2022-05-02

**Authors:** Annette Sofie Davidsen, Johanna Falby Lindell, Cæcilie Hansen, Camilla Michaëlis, Melissa Catherine Lutterodt, Allan Krasnik, Marie Louise Norredam, Susanne Reventlow

**Affiliations:** 1 The Research Unit for General Practice and Section of General Practice, Department of Public Health, University of Copenhagen, Copenhagen, Denmark; 2 Department of Nordic Studies and Linguistics, University of Copenhagen, Copenhagen, Denmark; 3 Section of Health Services Research, Department of Public Health, University of Copenhagen, Copenhagen, Denmark

**Keywords:** Language barrier, Interpreter, General practitioner, Consultation, Understanding, Ethics, Legal dilemmas

## Abstract

**Background:**

In 2018, an amendment to the Danish Health Care Act was passed making it a requirement for patients not proficient in Danish to pay for interpretation services in health care settings. Thereafter there has been a drastic decline in the use of professional interpreters, especially in general practice. We aimed to investigate the experiences of general practitioners (GPs) in establishing an understanding with these patients in consultations, without the presence of a professional interpreter.

**Methods:**

The study was qualitative, based on semi-structured interviews with nine purposively selected GPs. Analysis was by interpretative phenomenological analysis.

**Results:**

The GPs said that after the amendment was passed, the patients chose to almost exclusively use family members or friends as ad hoc interpreters, or they attended consultations with no interpreter present at all. The GPs experienced that the use of family interpreters caused specific problems, due to both their relationship with the patient and their lack of professional interpretation skills. If no mediator was present the GPs perceived the establishment of understanding as extremely challenging. This was particularly the case if patients had chronic conditions, mental or psychosocial problems or if cultural barriers were present. According to the GPs, the challenges were not exclusively restricted to a lack of language translation, but could also involve intertwined cultural barriers or social problems. The impairment in mutual understanding had different consequences, and led to poorer treatment at many levels in health care. The lack of access to a professional interpreter also presented the GP with ethical and legal dilemmas.

**Conclusions:**

The GPs experienced that the changes in interpretation provision for patients in health care had led to professional interpretation being almost absent from general practice settings for patients subject to the fee. This led to several communication challenges, insufficient understanding in consultations, and poorer treatment of these, often very vulnerable, patients. The situation could, however, also involve the risk of epistemic injustice. The GPs experienced the situation as very unsatisfactory; it both comprised their ability to exercise their professionalism and their ethical obligations and restricted their legal rights.

## Background

The number of migrants is rising worldwide [1], which means that an increasing number of patients who do not speak the local language challenge the health care systems in their host countries [2]. To ensure a mutual understanding in the interaction between patient and health care provider, a professional interpreter could be needed [3]. Due to budget cuts, however, access to such interpretation services in various countries is being restricted [1, 4]. Denmark is one example where this is the case. In 2018, an amendment to the Health Care Act was passed stating that immigrants who had lived in Denmark for more than three years must pay for an interpreter if needed in health care [5]. The reasons given were that this would motivate immigrants to learn the local language. Only in certain cases, e.g. where patients due to physical or mental disabilities are unable to learn Danish, can an exemption be granted [5, 6]. Since the introduction of the law there has been a considerable decrease in the use of professional interpretation services especially in general practice [7–9]. In this article we will examine how it affects consultations with patients when there is a language barrier and no professional interpreter is present. Our focus will be on general practitioners’ (GPs’) experiences.

Literature shows that language barriers lead to unequal access to healthcare and lower satisfaction in both patients and providers. This includes workplace stress, poorer communication and increased use of healthcare services [10]. Without professional interpretation the treatment is often deteriorated or delayed, and there is a risk of diagnostic and medication errors or lack of compliance [3]. Admission rates and use of emergency services are increased for these patients and they are subjected to more tests, so the immediate savings may increase health care costs later [11–13].

Challenges due to language barriers are considered particularly high in general practice [14]. The consultation there is a multi-task procedure that concerns both biomedical and lifeworld problems. The consultation often represents the patient’s first presentation of the problem to a health professional [14, 15], and failure to comprehend patients’ symptoms or problems may lead to wrong decisions [9].

Reviews have concluded that professional interpreters can improve quality of care with fewer errors in translation, greater satisfaction among both patients and practitioners, and a greater increase in clinical outcome than ad hoc interpreters [3, 16, 17]. However, the role of the interpreter is not necessarily tied to whether the interpreter is professional or ad hoc [18, 19]; patients may prefer family interpreters because they provide a different kind of support [17, 18, 20]. Particularly in uncomplicated situations, patients may show a preference for family members. They are able to trust and feel secure with their family, who is also easily available [1, 21]. Patients might also be suspicious of the professional interpreter’s ability to be objective, perhaps due to political conflicts; on the other hand they could feel ashamed to discuss some matters in the presence of family members [22–24]. However, family interpreters may also try to influence the consultation process [25]. Because they share the patient’s lifeworld they can shift the power balance in the patient’s favour and lead to greater inclusion of the patient’s lifeworld [26].

In all consultations with an interpreter present, either professional or ad hoc, it is challenging for doctors to convey empathy [19] and difficult to establish a trusting relationship [2]. Doctors have also been shown to be less affective when working with patients from ethnic minority groups [2, 9]. When a language barrier is present it demands greater communication skills from the professional [27]. Many immigrant patients are traumatized, having experienced torture or other abuses, or suffer from postmigration stress [28, 29]. Such psychological conditions have been shown to be especially challenging to deal with for the GP when there are language problems, and a stable relationship built on trust is essential [29].

Studies of GPs’ perspectives have mostly investigated their use of different types of interpreters [17, 30, 31], and how structural factors can impede the introduction of professional interpreters [32]. In primary care, it is much easier to make arrangements with an informal interpreter than with a professional; visits may occur at short notice and at various locations and times [14, 22]. A study has shown that GPs often try to ‘get by’ without interpretation [24]. Few studies have focused on the interaction in consultations between GP and patient without an interpreter present [33, 34]. These linguistic studies show a harder and more protracted interactional task for both patient and doctor; they also show that the patient cannot present symptoms in a way that fits into the ordered communication model with which the doctor is used to working. To our knowledge no studies have investigated the GPs’ experience of consultations with patients when there is a language barrier and no professional interpreter is available.

The aim of this article, therefore, is to explore how GPs experience communication with patients with low Danish proficiency without a professional interpreter, and which factors they perceive as either challenging or important for creating a common understanding with the patient.

## Method

The study was qualitative, with data material consisting of semi-structured in-depth interviews with nine GPs from two regions in Denmark. The study is part of a larger study on experiences related to the introduction of a patient fee for professional interpreters in Danish health care. The study also involves video-recorded consultations between the same nine GPs and patients with limited Danish proficiency, and interviews with some of these patients as well as interviews with patients selected in other ways. Results from the other part studies will be reported elsewhere.

### Participants

We invited GPs from a Danish website Sundhed.dk and from a list of tutors for medical students, and used snowball sampling. Nine GPs were purposefully selected aiming at variation in age, sex, seniority as a GP, and experience with the patient group. Four were men and five were women. The age range was 38–68 years (mean 54), and the seniority in practice varied between one and 31 years (mean 13.3). GP characteristics are reported in Table 1.

**Table 1:** Characteristics of participants

Participant no	Age	Sex	Seniority as GP, years	GPs’ self-reported load of immigrants in practice
GP1	46	Female	3	medium
GP2	65	Female	25	high
GP3	61	Female	17	high
GP4	68	Male	31	medium
GP5	45	Female	3	low
GP6	64	Male	24	low
GP7	43	Male	3	low
GP8	38	Female	1	medium
GP9	56	Male	13	medium

### Interviews

Semi-structured interviews were carried out at the GPs’ clinics, with the exception of one which was carried out in the GP’s home office with no other persons present. An interview guide was designed to ensure that all relevant topics were covered in relation to the GPs’ experiences with communication with patients with limited Danish proficiency and without a professional interpreter. The questions were open-ended and encouraged the informants to recount their experiences freely without including preconceived concepts. The interviews lasted 32–62 minutes (mean 50 min.) and were performed by two of the authors; seven by the first author and two by the second author. Three interviews were transcribed verbatim by the third author and the rest by a student.

### Analysis

Interpretative phenomenological analysis (IPA) was used for the structural analysis [35, 36], using both NVivo 11 and functions in Word 2016 to facilitate the analysis.

IPA relies on data from semi-structured interviews. The interviews do not strictly focus on description but may incorporate questions about the person’s attitude, beliefs, and general reaction on a topic; these are, nevertheless, always linked to lived experiences. The aim is to get as much detail as possible about the participant’s experience and to enter their lifeworld. The analysis is idiographic and longitudinal, with each interview being analyzed individually before looking at general features across the interviews. The analysis was carried out in five stages:

In the first step, we read and re-read the transcripts for meaning, making notes or memos about thoughts, reflections and observations in the left margin.

In the second step, we started identifying themes. Here patterns and themes were drawn out. This step is actually a thematic analysis, carried out in several rounds. First, we recorded initial themes using the right-hand margin. Chunks of meaning were coded as initial themes. The process was stepwise, so that the themes reached an increasing level of abstraction in the different rounds. In the last rounds, theoretical language started being used to capture the conceptual meaning but we still remained open-minded and tried not to impose meaning on the text. The aim was to see the world through the participant’s eyes and bracket off our own preconception.

In the third step, the themes were structured. The themes in the individual interviews were listed and relations between them were looked at; they were structured into clusters and hierarchies of meaning by generating superordinate and subordinate themes. Thereafter, the clusters were checked with the data to make sure that we did not go beyond the data or begin to theorize too quickly.

In the fourth step, a summary table of the themes and clusters was produced for the individual interview along with quotations to illustrate each theme and cluster.

The last step was integrating cases. We now made a table of master themes and constituent themes for the whole data set as a basis for the final writing up.

The first author carried out the primary analysis and thereafter the themes and the analysis were discussed among all the authors who were familiar with the data material. All quotations were translated from Danish to English by the first author and afterwards verified by a native English speaker who understands Danish.

## Results

The GPs said that after the amended act was passed, most of the affected patients could not afford to pay for a professional interpreter. Family members or friends were used as ad hoc interpreters instead, or patients tried to get by without an interpreter. Although the GPs had also experienced problems with the quality of professional interpreters previously, they described how the use of family members as interpreters might cause other specific problems; their relationship with the patient and their lack of professional training in interpretation led them to have quite a different role in the consultation. If no mediator was present and the interaction with the patient was direct, the GPs perceived the understanding as extremely challenging, especially when the consultation concerned complex situations, mental or psychosocial problems. The consultation was also experienced as challenged due to diverging health beliefs, and because these patients often had many other problems; these often eclipsed the health problems that were considered to be the subject of the consultation. The challenges were therefore not restricted to a lack of translation of language. The GPs said that the impaired mutual understanding had different consequences, leading to poorer treatment at different levels while also leaving the GPs in ethical and legal dilemmas.

Below the results will be described under the identified themes mentioned in Table 2.

**Table 2:** Identified themes

Themes	
1. Family interpreters hamper neutrality – but it is not black and white	
2. Doubt about the understanding – and improvised solution	
3. It’s not just language	
4. Derived consequences at many levels	
5. Relationship and trust promote understanding	
6. Squeezed between two opposing obligations	

### Family interpreters hamper neutrality – but it is not black and white

Overall, the GPs said that when family members acted as interpreters the interaction would be a conversation, either between the patient and the family member, and only briefly conveyed to the GP, or between the GP and the family member to the exclusion of the patient.

Sometimes the GPs experienced that the patient and the family member had a long exchange of sentences, which was translated to only one or a few words. The GP could also notice that the patient and the family member were talking loudly, with marked body language, without the GP understanding what was going on.

*When they start talking between themselves, perhaps a little more thoroughly between themselves or loudly, then you basically sit and think what is actually going on now, then sometimes you must, like, intervene and say: “well stop, what are you talking about?” (GP6).*

In such situations, the GP could feel left behind and having to fight to get the dialogue back on track. The GPs said that they could often be in doubt if the translation was correct or if something was omitted, especially when mental or social problems were concerned.

In some situations, the GPs experienced that the patients left out topics they thought their relatives should not hear about, or did not mention problems which might involve their relatives. In these situations, they felt it was difficult to get into depth with the patient’s situation and life story because it stopped at a certain point:

*There is something about him which I have never got a good grip of…something serious had happened at some point in his childhood in his home country…it was with a relative [as interpreter]…where we could like say well this was what I could get to know, and then there was a clear sign that we should not go any further. (GP7)*

Between spouses, in particular, the relationship could be experienced as asymmetric and the translation as defective. If the children of patients acted as interpreters, including adult children, the GPs experienced that they were often influenced by the family relationship. It was the GPs’ impression that these children’s neutrality conflicted with loyalty towards their parents, and that they were sometimes rehearsed by the family in advance or *“taken as hostages” (GP6)* to put pressure on the GP when social problems were involved and the GP was expected to write a certificate to the municipality:

*I think they are influenced also if the parents want to obtain some form of social benefit and have to present themselves in a certain light, then they rehearse with the children at home before going to the doctor … that’s how the story sounds at home: “you can see your mother is sitting over there in the sofa able to do nothing. That’s what we are going to tell the doctor”. (GP4)*

The GPs said that with family interpreters it was more difficult to stay focused on the patients’ health issues. The family interpreter might, however, also contribute information on the psychosocial situation of the family, leading to a more family-centred approach.

The GPs recounted that family members could also have the role as caretakers, being trusted by the patient and supporting them in the health care system. It might be meaningful, therefore, to use them as a mediator when the patient wanted it:

*There are some other factors that turn out to make sense as to why the patients want their family members to join them…something that has nothing to do with language or interpretation, that “here is somebody who likes me and will support me and understands a little better what it is like to go to the doctor, and [understands] the surrounding society”. (GP9)*

One GP had experienced a situation with a refugee, who he had known for many years and who had experienced serious torture. This had led to *“sexual thoughts, which were not quite ordinary”* (GP4). The patient could talk about this more easily in front of his son than he had been able to in front of a professional interpreter. This GP said that it was a case of balancing the benefits against the drawbacks in deciding which type of interpreter should be preferred, and that the solution was not black and white.

### Doubt about the understanding – and improvised solutions

All the GPs recounted that if the consultation was carried out without an interpreter they often felt in doubt whether a mutual understanding was established. To ensure a better level of understanding, the GPs said that they repeated their explanations and the treatment plans many times; they made drawings to explain things, wrote down how patients should take the medication, and they summed up at the end of the consultation. When summing up, however, they often became aware of how little the patients had understood, even though they had pretended to understand:

*I repeat really many things, say it five times or so…I try to sum up at the end of the consultation, and that is also where I sometimes find out, oh they have not understood what I say…actually it is often when I sum up that I find out how little they [have understood] – although they sit saying yes, yes and I have written down and I have.. (GP1)*

This made the GPs feel that sometimes it was *“a great deal of a guessing competition” (GP6)*. Some GPs considered that the patients pretended to understand to be polite, possibly because they had a more traditional view on the doctor-patient relationship with no equal interaction.

The GPs recounted that they had to use simple language where the possibilities for rephrasing were limited. This simplified language was without nuances implying that *“you never feel that you get to the bottom of things”* (GP5).

The GPs said that medical explanations were difficult. In the case of a metabolic disorder, for example, the patient would perhaps not require medical treatment at that very moment but would need to understand the importance of specific self-care measures, observation of symptoms and control. Without an interpreter it was difficult to know if the GP’s explanation was understood correctly:

*If for example, I have to tell people about a metabolic disorder…and why they cannot feel it and all those things, well this medical story is sometimes so complicated and it is important that it is understood correctly. (GP3)*

Chronic conditions, especially lifestyle diseases such as diabetes, created particular problems for the GPs. They felt that it was difficult to explain to patients that this was a lifestyle condition and that the reason for the treatment was to prevent future complications and not just to treat symptoms:

*There is so much information about the chronic conditions, which is really difficult to give when there is an impaired linguistic understanding. It is very much about that they understand the reason for their treatment if they should follow the treatment; that they understand the meaning of it. (GP8)*

Chronic pain conditions were considered particularly difficult to handle, because the GPs felt that they did not succeed in explaining the condition and reaching a common understanding about what could be done. The GPs experienced these situations as unsatisfactory and even if they felt that their relationship with patients was good, some things did not work:

*…[I get] a feeling that I have not done the work properly and I wonder if I have understood the problems correctly, that is unsatisfactory in some way. (GP5)*

To ease the understanding, GPs sometimes used other tools such as calling a family member on speakerphone, using Google Translate, or speaking English. Google Translate did not work for illiterate patients, and English only worked for patients who had a certain English proficiency. In addition, the GPs felt that their own English was not nuanced enough. Others gave patients the Latin names of diseases, which they could then ‘Google’ in their native language. All these tools were, however, considered by GPs to be an improvised and poor solution.

### It’s not just language

The GPs said that many of these patients were highly stressed or traumatized, which prevented them from learning the Danish language. Many had complex social problems, which were intertwined with their limited linguistic understanding. Other patients had very little previous schooling, and the GPs considered that this made it difficult for them to understand the seriousness and long-term consequences of chronic conditions and the need of prophylactic medication.

The GPs recounted that the municipality might provide courses about lifestyle, but they were mostly in Danish. If these courses were conducted in Arabic, as they occasionally were in the larger municipalities, they were either not ran for a long enough period, the patients did not fit into the predetermined boxes, or they had other problems that eclipsed the chronic condition and deprived them of the motivation to participate:

*If you happen to fit into one of those boxes, then they [the municipality] actually have courses with Arabic speaking teachers…but the problem is that they [the courses] do not continue, and there are really many patients who do not fit in, and where their real problems are not at all about the diseases for which they get rehabilitation, it’s some socioeconomic and heavily functional problems, about pain conditions. (GP9)*

Part of the problem was considered linked to cultural issues. The GPs said that some patients with diabetes understood the condition as it was understood in their home country, where many of their family members had diabetes and where the treatment did not include lifestyle interventions:

*I think he knows diabetes in the understanding of his own culture…but I do not think he has understood what I have said, for I do not think he can maintain a diabetes friendly lifestyle. (GP5)*

The GPs experienced that for some patients the chronic conditions were overshadowed by other, more existential, problems and problems related to their residence permit:

*…rather than what I thought we were going to talk about, something as trivial as their chronic diseases, which do not preoccupy them at all, a recurrent focal point, which of course preoccupies them existentially is if they can be thrown out of the country. (GP9)*

### Derived consequences at many levels

The GPs recounted that often the patient did not understand communications, either by phone or electronically, from specialist departments to which the GP had referred them and consequently they missed their appointments and their case was closed. The patients might also misunderstand details of where they were referred to; they might, for example, mistake a physiotherapist for a rheumatologist, then terminate their treatment after being informed by the former that they would need to pay a patient fee. There were also cases where patients had been referred to an examination at the hospital and the examination involved different elements; the patient did not understand this, left the department after the first part of the examination, and so the GP had to make a repeat referral:

*She came to have an X-ray and after that an ultrasound of her shoulder, and that is in two steps, but when the X-ray was made she simply had not understood that she should also have an ultrasound examination, and then she had just left…and that [the ultrasound] was actually the most important examination. (GP6)*

Language problems also led to difficulties in looking at a condition with fresh eyes. If it was difficult to obtain a symptom description during their consultation and the patient had visited emergency clinics and other doctors previously, the GP used the written material from the preceding examinations. This led to the GP being in danger of *“placing themselves in the slipstream” (GP5)*. A GP recounted a case where the patient, who had had many consecutive visits with the same symptoms, was at last admitted to hospital where it turned out that she suffered from Covid-19.

The GPs also experienced problems in relation to medication. Patients had difficulties recognizing that a substituted medication contained the same active ingredient when the name and the packaging differed. This meant that the GPs often had to prescribe the more expensive original drug to avoid medication errors. Patients also had difficulties understanding regimes for stepping up medication, even though the GPs wrote it clearly on notes. The GPs said that they had to offer extra control consultations to ensure correct medication, for their own sake as well as the patients’:

*There is an endless row of communication challenges…the only way that I manage it somewhat unhurt is when I for the first, second, third, fourth time force them to bring their medication and then we go through it on the desk, then we avoid the mistakes, otherwise it is impossible to communicate about. (GP9)*

The GPs expressed frustration about not helping patients as well as they could if there had been an interpreter present. Particularly with regard to lifestyle conditions, they said that they often had to give up, because *“you cannot push those buttons” (GP4)*. As for pain conditions, the GPs felt that there were great difficulties in avoiding the onset of pain-killer dependency because they could not establish a dialogue with the patient about the mechanisms giving rise to the condition and other non-medical treatment possibilities:

*As doctors we want to help and we want to do something, and the risk is that then they come and argue strongly for medicine because you must be able to make the pain disappear, and then you get down a stupid road. (GP5)*

### Relationship and trust promote understanding

A few GPs talked about the importance of a trustful relationship. Some said that as their knowledge of the patient gradually developed it promoted understanding, making their work easier and more satisfactory. In getting that knowledge and building a relationship it was necessary to have a good mediator, which might be a family member or a friend:

*My sighing is less pronounced when I know the patients…how do they look when they are healthy, know how they look when they are ill, know the relatives…it is more difficult to get a relationship if you do not have some interpreter. (GP7)*

Another GP talked about the importance of being patient-centred in order to develop a trustful relationship. He also said that one cannot avoid having prejudices but one should work professionally to realize them. He said that due to their vulnerability, it might be difficult for these patients to contact the health care system and to trust the professionals; it was possible over time, however, to build a trustful relationship which could make understanding easier and break down the prejudice:

*I think we are prejudiced in every meeting with other people but many patients who need interpretation are so vulnerable in all ways that it is already a little difficult, at least to begin with, to go to the doctor and open up until they find out that actually the doctor wishes them well and then all prejudices break down mutually when the relation is gradually built up. (GP9)*

### Squeezed between two opposing obligations

All GPs experienced that it was an ethical problem to not have access to professional interpretation services. The GPs realized that both illness and cultural differences put great strain on these patients, and it was the GPs’ experience that the politically-motivated introduction of a patient fee for interpreters just worsened the patients’ conditions and led to increased care inequality. The GPs expressed a high level of moral indignation and they did this in an indignant language:

*It [patients paying for interpretation services] is completely daft, it does not make any sense at all…you should do just the opposite, and as a doctor you are put in some specific dilemmas. (GP4)*

*I experience that it is some political decisions that mean that you should trip them up, because then fewer people want to go to Denmark, and those who are there they probably hurry home, but it is hellishly inhuman. (GP6)*

Legally it was the GPs’ responsibility to procure the services of a professional interpreter when there were language problems. The GPs, however, also felt a responsibility towards the patient and did not feel that they could book an interpreter, which the patient would have to pay for, without asking the patient:

*I have the treatment responsibility and if I do not succeed in asking detailed questions then it is my responsibility if the patient does not get the right treatment and it is my responsibility to make myself understood and it is my responsibility to ensure that the patient has understood what I say and also that I have understood what they say, so there are a lot of dilemmas with which we compromise…that is really scary. (GP5)*

The GPs felt that their legal rights were not secured because they were squeezed between two opposite considerations; their ethical obligations to the patient and the Hippocratic Oath, and their legal obligations:

*I think that our legal certainty is bad and we all the time move at the edge of law so that I think that in a lot of what I am doing I constantly put myself in some danger…you walk on a tightrope. (GP5)*

Another GP said that he felt stuck in the middle of what was political and what was medical. The politicians had *“taken health as hostage” (GP4)* and the doctors were taken as *“political hostages” (GP6).*

*You have the choice of which law to break, because you cannot avoid breaking one of them, that is actually not very pleasant. (GP6)*

## Discussion

The GPs described how the introduction of the fee had led to patients using family members or friends instead of a professional interpreter, or to seeing the GP on their own. Using family or friends as interpreters caused problems with confidentiality, important issues not being mentioned, and a power imbalance; the family members were sometimes too entangled in the patient’s problems themselves to act for the good of the patient. They were not always experienced by the GP as being neutral, for example in situations where the family wanted the patient to receive some social benefit or if asymmetric gender roles were enacted, as also shown in the literature [25, 29]. The consultations with family interpreters became a conversation with three interlocutors, leading to the doctors feeling a loss of control, as also found by Zendedel et al. [1], and, in line with Brisset et al. [19], to a change in the power dynamic in the consultation. However, although they preferred professional interpreters, the participants found that there was no clear-cut answer as to whether to use family members, because in addition to interpreting the family members often also had other roles. The patients might prefer a family member because they trusted them, or they could offer support or were easily available for unplanned visits, a finding consistent with the literature [1, 2, 17, 18, 22, 23, 25].

As also found in the literature [10, 18, 28], the GPs in our study felt that they did a bad job in many of these consultations, and communication without the presence of a professional interpreter was perceived as a great challenge. This was especially so in complex diseases such as chronic lifestyle conditions and mental disorders where the problems often went beyond language problems. The GPs felt they had to compromise their professionalism and that the patients therefore received poorer treatment. When complicated medical explanations were needed, the GPs preferred to use a professional interpreter as also shown in other studies [2, 9, 30, 37]. Nevertheless, the presence of a professional interpreter was not seen as a blanket solution. This suggests that the choice of type of interpreter should be tailored to the preferences of the individual patient.

Some of the participants emphasized the importance of the doctor-patient relationship, of gradually developing an increased knowledge of the patient, and the importance of being patient-centred [38]. They said that the development of this relationship demanded a good mediator who, as reported in other studies [18, 27], could be a family member or friend. A review has shown that a trusting and continous relationship is essential for effective healthcare [29] while another study has found that language barriers lead to significant impediments in establishing an effective doctor–patient relationship [9]. This means that these patients are in a vulnerable position due to language incompatibilities [28, 39] something that according to our participants greatly affected the provision of health care.

Communication is required to gain understanding of the reason for the patient’s visit, for an exchange of information about symptoms, the presumed diagnosis, and so on [27]. Our participants often felt that in spite of their great efforts to explain things they did not reach mutual understanding with the patients. Literature has shown that this may be a question of linguistic as well as cultural understanding; different cultural approaches to illness and health care concepts, and difficulty understanding often complex health care systems play a role [9, 28, 29]. According to our participants, impaired linguistic understanding influenced treatment at many levels. The treatment of chronic and complex conditions in general practice was impaired and there was a risk of medication errors. In addition, the patients did not always understand communication from hospitals or other services to which they were referred by the GP. They therefore did not show up and their case was closed. The GPs did not get to know the details of the patients’ background and cultural understanding; and due to the language barrier the GPs had to refrain from exploring these issues. This in turn led to them conveying less empathy towards such patients, a finding echoed in other studies [2, 19].

A GP talked about his awareness of his own prejudices and his work with overcoming them. He expressed the need for understanding the patient’s lifeworld and existential challenges in a patient-centred way [38]. The literature has stated that a significant part of the language barrier problem relates to the more covert role that language plays in meaning-making processes; because the dialogue is inevitably situated in a particular cultural and linguistic setting, insensitivity to different conceptualizations of health and illness in different languages can result in what Peled [40] has called hermeneutical epistemic injustice. Some GPs mentioned that they considered some of these patients to be less intelligent than average. This could be a way the language barrier unjustly influenced the perception of the patient, and an issue that could call for a greater awareness of the need of epistemic humility [40].

Despite acknowledging that several factors influenced their ability to help their patients, such as cultural differences and the complexity of the patients´ problems, the GPs felt a professional insufficiency when they could not treat the patients according to their own standards, the national guidelines and the politically influenced accreditation.

The GPs remarked that their legal rights were not secured with the amendment on fees for professional interpreting, because they were squeezed between two opposing obligations. As doctors they had to act in accordance with not only the Hippocratic Oath; to become doctors they had also signed a specific Danish version of the oath, which states that a doctor must treat all patients equally, without taking into account or being influenced by patients’ social, economic or ideological positions [41]. On the other hand, the doctors are obliged by law to ensure that each patient is properly informed and that an interpreter is present if there are language problems; this is the case even if they know that the patients cannot pay the fee. As the Minister of Health said:

*“Nothing has changed at this point after the fee for an interpreter has been introduced. It is the doctor’s obligation to secure that the patient is properly informed, and therefore the doctor must book an interpreter, if there are problems with understanding. The patient shall not pay for the interpreter before the treatment, and the subsequent bill to the patient will be collected according to common practice.”* (https://sundhedspolitisktidsskrift.dk/nyheder/2884-heunicke-laegerne-har-ansvaret-for-fejlbehandlinger-pa-grund-af-manglende-tolk.html) (Author’s translation. “Common practice” means debt collection).

This situation made the GPs express great frustration and the feeling that they were taken as hostages by the politicians. Consultations with language barriers thus represented both an ethical and legal dilemma for the GPs.

Many of the problems seen in these patients are an intensification of the same problems that have been recognized in other vulnerable patients [42, 43]. This pertains both to the complexity of the problems, involving social as well as existential issues, and to the effects of low health literacy and problems navigating the health care system [39, 44]. In this way these immigrant patients can be considered a critical case in health care [45, 46].

The study has several possible limitations. There were only nine participants. There was, however, an equal gender distribution, the participants were of different ages and seniority, and they were from two different regions. Despite being selected with variation with regard to several key characteristics, they described facing nearly identical problems in consultations with patients where there was a language barrier; they also all felt the same ethical and legal dilemma when treating these patients. This makes us believe we have reached information power [47], and that the findings could be transferrable both to other GP consultations in Denmark and to countries with similar primary health care systems.

## Conclusions

GPs experienced that the patient fee for professional interpretation in health care had led to the use of these services being almost completely absent in general practice for patients subject to the fee. This led to several communication challenges and insufficient understanding, both when ad hoc interpreters were present and when no interpreter was present and had consequences for patients at many levels of health care. A trustful relationship was considered helpful but the establishment of it demanded a mediator. The interaction also involved a risk of epistemic injustice. The GPs experienced the situation as unsatisfactory, both comprising their professionalism and leaving them in ethical and legal dilemmas. These patients were seen as especially vulnerable due to the complexity of their problems; and the GPs found language barriers exacerbated cultural differences and made it more difficult to help the patients. This could be considered an intensification of problems recognized in other vulnerable patients. Therefore, immigrant patients can be considered a critical case in health care.

## Data Availability

The data used and analysed during the current study are stored on a secured drive at the University of Copenhagen. Although the interview transcripts have been anonymised as regards names and locations the participants could possibly be identified, for which reason the transcripts cannot be made publicly available. The anonymised data are, however, available from the corresponding author upon reasonable request.
